# Particle bombardment-assisted peptide-mediated gene transfer for highly efficient transient assay

**DOI:** 10.1186/s13104-023-06320-3

**Published:** 2023-04-06

**Authors:** Mitsuhiro Kimura, Akira Endo, Yozo Nagira, Takeshi Yoshizumi

**Affiliations:** 1 Faculty of Agriculture, Takasaki University of Health and Welfare, Takasaki-shi, Gunma, Japan; 2 Agri-Bio Research Center, Agri-Bio & Supplement Research Laboratories, Kaneka Corporation, Iwata, Shizuoka Japan; 3 Present Address: Faculty of Agriculture, Kyushu University, Fukuoka, Japan

**Keywords:** Particle bombardment-assisted peptide-mediated gene transfer (PBPT), DNA-peptide complex, Nanoluc

## Abstract

**Objective:**

A centrifugation-assisted peptide-mediated gene transfer (CAPT) method was recently developed as an efficient system for gene delivery into plant cells. However, the gene transfer efficiency of CAPT into plant cells was not entirely satisfactory for detecting transient expression of a transgene driven into mitochondria. Here, we report a new gene delivery system using a method called particle bombardment-assisted peptide-mediated gene transfer (PBPT).

**Results:**

We investigated various parameters of the PBPT method to increase transient gene expression efficiency in *Brassica campestris*. The optimal conditions for PBPT were a single bombardment with gold particles coated with a DNA‒peptide complex (6 µg of DNA and 2 µg of peptide) at an acceleration pressure of 5 kg/cm^2^ and a target distance of 12.5 cm. Moreover, bombardment under the optimal conditions successfully transferred the transgene into the cells of other plant species, namely *B. juncea* and tomato. Thus, we developed a PBPT method for highly efficient delivery of a DNA‒peptide complex into plant mitochondria.

**Supplementary Information:**

The online version contains supplementary material available at 10.1186/s13104-023-06320-3.

## Introduction

Mitochondrion is an essential cellular organelle for catalyzing ATP synthesis by oxidative phosphorylation in eukaryotes [1]. Compared to canonically mammalian mitochondrial genomes, higher plant mitochondria have larger genomes and greater numbers of mitochondrial genes [2]. In several plant species, the cytoplasmic male sterility (CMS)-associated gene is located on the mitochondrial genome, and the CMS trait is valuable in crop breeding [3]. In conventional plant breeding, the CMS trait can be introduced only from crossable plant species with CMS-associated genes. Thus, theoretically, the introduction of an exogenous CMS-associated gene could be accomplished by transmitochondrial technology in all plant species.

On the other hand, transmitochondrial technologies have not been established in higher organisms [4]. Recently, we developed a system for gene delivery to plant mitochondria by using a fusion peptide composed of polycationic peptides with mitochondrial targeting signals (cytocox-KH) and successfully integrated a transgene into a plant mitochondrial genome [5, 6]. We also developed the centrifugation-assisted peptide-mediated gene transfer (CAPT) method as a high-throughput means of transferring DNA‒peptide complexes to plant organelles [7].

It has been reported that many mitochondria exist in plant cells; for example, a single onion epidermal cell contains more than 15,000 mitochondria [8]. Since mitochondria are more numerous than nuclei and chloroplasts (80 to 120 chloroplasts per Arabidopsis mesophyll cell [9]) in a plant cell, it is necessary to introduce many complexes into plant cells. In the present study, we developed a method called particle bombardment-associated peptide-mediated gene transfer (PBPT) that introduces complexes into plant cells more efficiently than the CAPT method.

## Materials and methods

### Plant materials and growth conditions

*B. campestris* (cv. Kyoto Fushimi Kanzaki Hanana), *B. juncea* (cv. Coral Reef Plume), and tomato (cv. Momotaro) seeds (all purchased from Takii Seed, Kyoto, Japan) were treated with 70% EtOH for 1 min, followed by 20% bleach for 15 min, then rinsed 3 times with sterile distilled water. The sterilized seeds were placed on germination medium (GM) containing half-strength Murashige and Skoog medium [10] with 10 g/L sucrose and 2.5 g/L Phytagel (Sigma-Aldrich, St. Louis, MO, USA) at pH 5.8. Seeds were germinated under a 16 h light/8 h dark condition at 30 °C (*B. campestris*) or 25 °C (*B. juncea* and tomato). After 7 days, the cotyledons of *Brassica spp.* and the hypocotyls of tomato were used as explants.

### Plasmid construction

pGWB-Prrn18: Nluc was constructed in the 35S-Nluc-TNOS backbone [11]. This plasmid contains the *NLuc* gene [12] driven by Prrn18. Prrn18 is the promoter region of the gene coding for mitochondrial *18S ribosomal RNA* (*rrn18*) and probably activates strong transcription [13]. The DNA fragments containing Prrn18 was amplified using the primers, SacI-rrn18 F (5’-GCGAGCTCCGGAAGTAGCGCGACAAAGAG-3’, SacI site is underlined) and KpnI-rrn18 R (5’- GCGGTACCAACTCTTCTTTTGAGTATGAT-3’, KpnI site is underlined) from *Nicotiana tabacum* mitochondrial DNA as a template. The PCR product of Prrn18 was inserted into the SacI-KpnI region of the 35 S-Nluc-TNOS vector to replace CaMV 35 S promoter (Fig. S1).

### Particle bombardment of DNA‒peptide complex

To prepare the DNA**‒**peptide complex, a plasmid, pGWB-Prrn18: Nluc, and a peptide, cytocox-KH (amino acid sequence: MLSLRQSIRFFKKHKHKHKHKHKHKHKHKH), were mixed by the method described previously [14, 15]. The DNA‒peptide/gold coating procedure was carried out as followings: 10 µL aliquots of gold particles (Tanaka Kikinzoku Kogyo, Tokyo, Japan; 0.3–2 μm in diameter, 60 mg/mL) were coated with the complex mixture (6 µg of DNA and 2 µg of peptide, 10 µL of 2.5 M CaCl_2_, and 4 µL of 0.1 M spermidine) by vortex vigorously. The mixture was incubated at room temperature for 60 min and centrifuged to collect the gold particles, which were then washed with 100 µL of 70% EtOH and resuspended in 15 µL of 99.5% EtOH. Fifteen microliter solution was used per bombardment.

The particle inflow gun and general bombardment methods were described previously [16]. Seven cotyledons of *B. campestri*s, 10 cotyledons of *B. juncea*, or 14 hypocotyl segments of tomato, respectively, were placed in the center of a 9 cm petri dish containing 20 mL of 1.5% agar plate. The cotyledons of *B. compestris* was bombarded at various target distances, DNA‒peptide complex loads per bombardment, numbers of bombardments, and acceleration pressures by nitrogen gas (Table 1). *B. campestris* was suitable for sample preparation and was used for optimize the parameters of PBPT method. The cotyledons of *B. juncea* or the hypocotyl segments of tomato was bombarded using the optimal conditions determined by *B. campestris*. The explants were transferred to GM and were incubated in the dark at 30 °C. After 1 day, the bombarded samples of 4 cotyledons of *B. campestris*, 6 cotyledons of *B. juncea*, or 7 hypocotyl segments of tomato were homogenized in GUS/LUC buffer [17] with 5.0 mm Stainless Beads (Biomedical Science, Tokyo, Japan) at 1,100 rpm for 30 s using Shake Master (Biomedical Science). The homogenized solutions were centrifuged at 10,000 xg for 10 min, and the luciferase activity of the supernatants was measured with the Nano-Glo Luciferase Assay System (Promega, Madison, WI, USA) analyzed on a GM2000 GloMax Navigator Microplate Luminometer (Promega). The total protein in each sample was quantified by the Bradford assay (XL-Bradford KY-1040, Apro Science, Tokushima, Japan). The luciferase activities were evaluated in relative light units (RLU) per milligram of protein.

**Table 1:** Particle bombardment parameters tested independently for the optimal conditions

Parameters	Conditions
Target distance (cm)	6.2, 9.6, 12.5^†‡^
DNA-peptide complex load per bombardment	x0.5, x1 (6 µg of DNA and 2 µg of peptide)^†‡^, x2, x3
Number of bombardments	1^†‡^, 2
Acceleration pressure (kg/cm^2^)	1, 2^†^, 3, 5^‡^, 7

^†^Standard conditions

^‡^Optimal conditions

### Transfer of DNA‒peptide complex by CAPT method

The CAPT method was performed as described previously [7]. Four cotyledons of *B. campestris* were added to 500 µL of the solution containing the complex mixed with 15 µg of DNA and 5 µg of peptide, followed by centrifugation at 10, 000 xg for 60 s. The luciferase activities were evaluated by the methods described above.

### Statistical analyses

All statistical analyses were performed by EZR software [18]. Statistical significance was determined using Mann–Whitney U-test. All values are expressed as mean ± standard error (SE).

## Results and discussion

**PBPT method compared to CAPT in*B. campestris***.

**Fig. 1:**
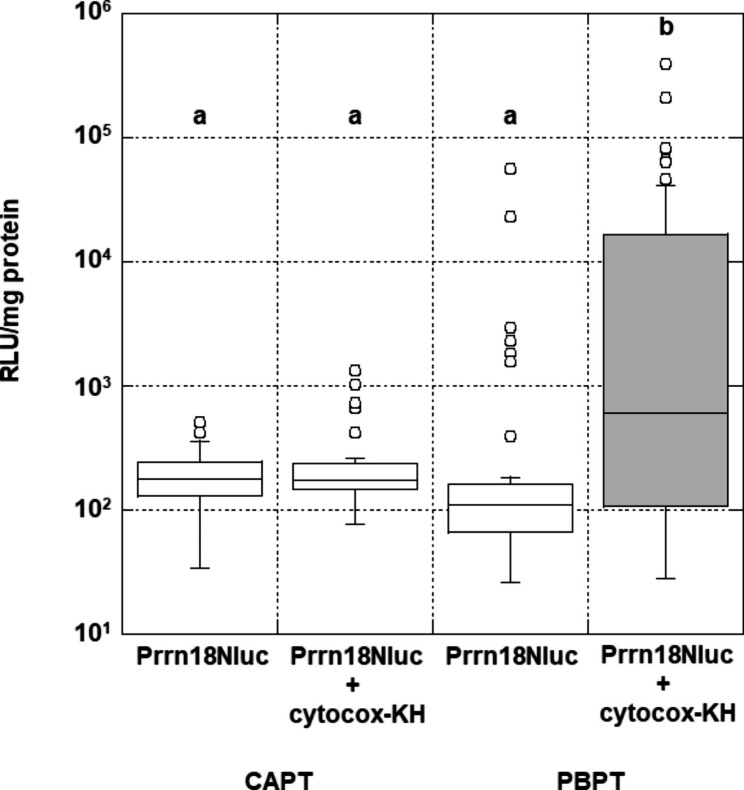
Transient expression of the Nluc gene in *B. campestris* transformed by CAPT and PBPT.

To develop the PBPT method, a reporter construct designated as pGWB-Prrn18: Nlu encodes Nano luciferase (Nluc) [12] under the control of tobacco mitochondrial *rrn18* promoter (Prrn18). The cotyledons of *B. campestris* were bombarded once with gold particles coated with either pGWB-Prrn18: Nluc only or a complex of pGWB-Prrn18: Nluc and cytocox-KH at an acceleration pressure of 2 kg/cm^2^ and with a distance of 12.5 cm between the syringe filter and the target plate. These parameters were used as the standard conditions (Table 1) to evaluate each parameter separately (n = 4 × 12). The gene transfer efficiency by bombardments with the DNA‒peptide complex showed a 5.4-fold higher median in comparison with that of DNA (*P* < 0.0001, Fig. 1). This result suggested that the particle bombardment method transferred a small amount of exogenous DNA solely to mitochondria. Using the complex with cytocox-KH, the median gene transfer efficiency of the PBPT method (*P* = 0.0009) was 3.5-fold that of the CAPT method (Fig. 1) [7]. Because Cytocox-KH does not contain a cell-penetrating peptide, it has a poor ability to permeate plasma membranes [5]. In the CAPT method, the DNA‒peptide complex can accumulate only on the plant cell surface and not actively penetrate plant cells [7]. Using particle bombardment, gold particles coated with the DNA‒peptide complex can easily penetrate plasma membranes [19]. Because their enables high permeability of plasma membranes, the PBPT method provides highly efficient delivery of complexes into plant cells.

### Effect of target distance in ***B. campestris***

Our inflow gun can be adjusted to three target distances: 6.2, 9.6, and 12.5 cm. We optimized the target distance based on the standard conditions (Table 1). The target distance is an important parameter for spreading gold particles with the DNA‒peptide complex over a target area [20]. Nluc expression bombarding a target distance of 12.5 cm is significantly greater than that of 6.2 cm (P = 0.0153, Fig. S2). No significant difference in Nluc expression was observed between 6.2 and 9.6 cm or between 9.6 and 12.5 cm (Fig. S2). Thus, the target distance of 12.5 cm is optimal.

### Effect of DNA‒peptide complex load per bombardment in ***B. campestris***

To identify the optimal conditions, the amount of DNA‒peptide complex is also an important bombardment parameter. We evaluated the DNA‒peptide complex load per bombardment. To prepare 8 µg of DNA‒peptide complex under standard conditions (Table 1), 6 µg DNA was mixed with 2 µg peptide. A total of 8 µg of the complex per bombardment achieved the highest Nluc expression (Fig. S3). In previous studies, particles coated with large amounts of DNA had low membrane permeability due to particle aggregation [21], and a high concentration of cationic peptide induced cytotoxicity in plant cells [14]. These results suggested that the expression levels induced by 16 and 24 µg of DNA‒peptide complex per bombardment were remarkably decreased.

### Effect of the number of bombardments in ***B. campestris***

The cotyledons *of B. campestris* were bombarded once or twice to analyze the efficiency of Nluc expression. Several reports indicated that multiple bombardments affected gene transfer efficiency in various plant species [21–23]. Nluc expression induced by two bombardments was significantly lower than that induced by one bombardment (P = 0.0100, Fig. S4). Multiple bombardments of target tissues may cause cytotoxicity induced by cationic peptide [14] and mechanical damage by gold particles [24]. Thus, a single bombardment is optimal.

### Effect of acceleration pressure in ***B. campestris***

Acceleration pressure is an important parameter for estimating mechanical damage to target plants. To achieve high gene transfer efficiency without mechanical damage by gold particles, we optimized acceleration pressure by nitrogen gas (1, 2, 3, 5, and 7 kg/cm^2^). The highest Nluc expression was observed at 5 kg/cm^2^ nitrogen pressure, and the 1 kg/cm^2^ nitrogen pressure had the lowest gene transfer efficiency (Fig. S5). Each explant has different characteristics, such as size, shape, thickness, and surface structure. Therefore, the acceleration pressure might be necessary to optimize depending on each explant [25]. These results demonstrated the optimal conditions for the bombardment of *B. campestris* cotyledons.

### Optimized bombardment conditions in ***B. juncea*** and tomato

**Fig. 2:**
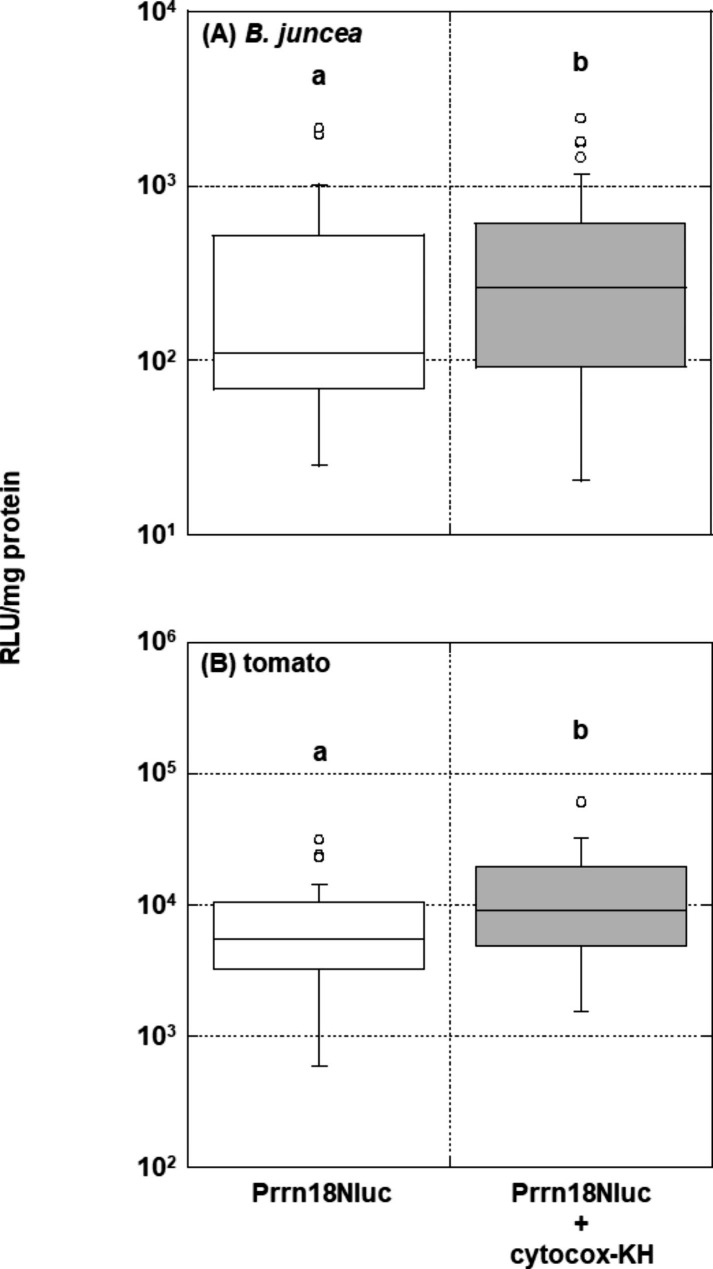
Transient expression of the *Nluc* gene in *other plant species* bombarded by the optimal conditions

To validate the PBPT method for use with other plant species, DNA‒peptide complexes were introduced into cells of *B. juncea* and tomato using the optimal conditions determined above. The Nluc expression levels in both plants were significantly higher than those in the respective controls (2.4-fold, P = 0.0293; Figs. 1 and 2A.7-fold, P = 0.0028; Fig. 2B. Customizing the parameters (e.g., acceleration pressure) for each plant species might enhance the PBPT method’s efficiency at introducing DNA‒peptide complexes into plant cells.

In the PBPT method, a majority of the DNA‒peptide complexes hit to cytosol are subsequently delivered to mitochondria by a function of mitochondrial targeting signals of Cytocox-KH [5, 6]. By conventional particle bombardment method, the plastid transformation of several crops has been reported [26], however, the mitochondrial transformation has not [27]. Despite the success of DNA delivery to mitochondria, the transmitochondrial plants have not been produced using Cytocox-KH [5, 6]. The CAPT method was developed to enhance DNA delivery using an artificial peptide fused with cell-penetrating peptide (CPP) for nuclear transformation [7]. We expected that a combination of Cytocox-KH and the CAPT method deliver a large amount of DNA to mitochondria. However, the CAPT method was insufficient for DNA delivery with Cytocox-KH lacking CPP to penetrate the cell membrane (Fig. 1). The PBPT method overcame the problem of Cytocox-KH showing less penetration of the cell membrane. Similar to transplastomic plants, homoplasmic transmitochondrial plants may produce more recombinant proteins than heteroplasmic plants [28]. Therefore, the efficient transformation of plant mitochondria by the PBPT method will require increased efficiency of the introduction of DNA‒peptide complexes into plant mitochondria.

## Limitation

Although we introduced transgenes into the mitochondrial genome by the optimized PBPT method and homologous recombination, we could not detect the transgene integration by Southern blot analysis. Since a selectable marker for mitochondrial genome transformation has not been developed, a transmitochondrial plant has not been produced yet. It is unclear whether Nluc expression in this study depends only the expression of Prrn18: Nluc in mitochondria, as we have not demonstrated that no cytocox-KH is delivered to the nucleus or chloroplast. In addition, it is unknown whether the PBPT method is applicable to monocots, such as rice, wheat, and barley.

## Electronic supplementary material

Below is the link to the electronic supplementary material.

Supplementary Material 1

Supplementary Material 2

Supplementary Material 3

Supplementary Material 4

Supplementary Material 5

## Data Availability

The data of the current study are available from the corresponding author (M. K.) on reasonable request.

## Abbreviations

PBPT: Particle Bombardment-assisted Peptide-mediated gene Transfer
Nluc: Nanoluc
CAPT: Centrifugation-Assisted Peptide-mediated gene Transfer
