# Supplementary material for: Particle bombardment-assisted peptide-mediated gene transfer for highly efficient transient assay
Source: BMC Res Notes. 2023 Apr 6;16:46. doi: 10.1186/s13104-023-06320-3 (PMC10080836; doi:10.1186/s13104-023-06320-3)
Supplement: Supplementary file 1 — Supplementary Material 1 [file 13104_2023_6320_MOESM1_ESM.pdf]

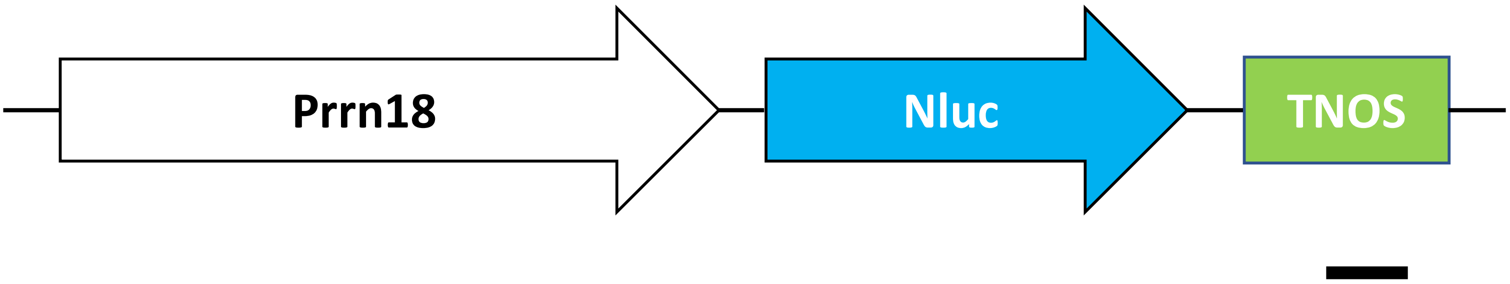


**Fig. S1 Schematic representation of pGWB-Prrn18: Nluc used in this study. Prrn18, rrn18 promoter region from *Nicotiana tabacum* mitochondria; Nluc, luciferase from the deep-sea shrimp (*Oplophorus gracilirostris*); TNOS, Nopaline synthase terminator from *Agrobacterium tumefaciens*. bar = 100bp.**
