# Supplementary material for: Particle bombardment-assisted peptide-mediated gene transfer for highly efficient transient assay
Source: BMC Res Notes. 2023 Apr 6;16:46. doi: 10.1186/s13104-023-06320-3 (PMC10080836; doi:10.1186/s13104-023-06320-3)
Supplement: Supplementary file 5 — Supplementary Material 5 [file 13104_2023_6320_MOESM5_ESM.pdf]

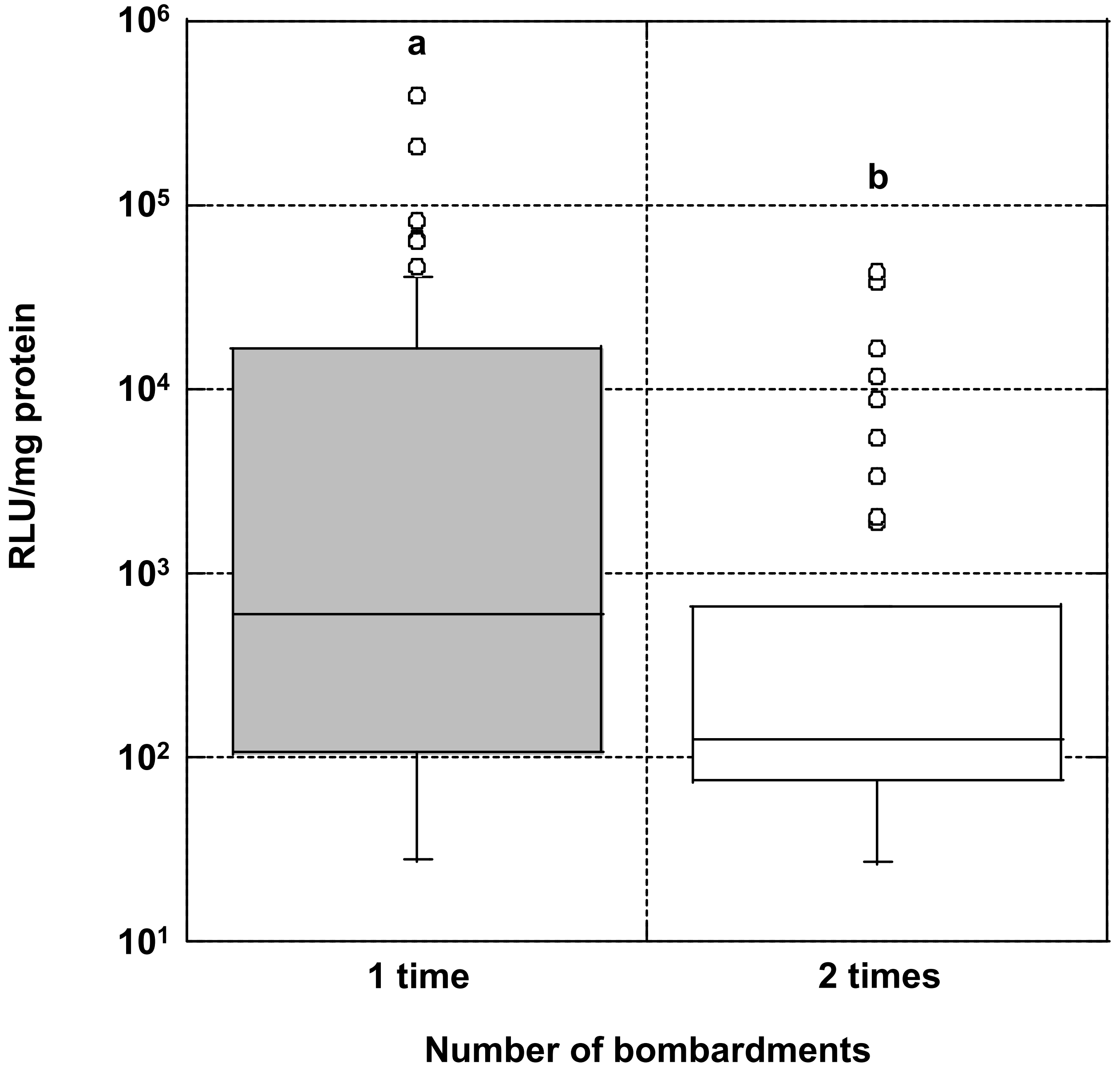

Fig. S4 Effect of number of bombardments on transient expression of the *Nluc* gene. Data are presented as the means ± SE from four independent biological replicates (n=4x10). Different letters above the bars represent significant differences ($P<0.05$).
